# Higher maternal leptin levels at second trimester are associated with subsequent greater gestational weight gain in late pregnancy

**DOI:** 10.1186/s12884-016-0842-y

**Published:** 2016-03-22

**Authors:** Marilyn Lacroix, Marie-Claude Battista, Myriam Doyon, Julie Moreau, Julie Patenaude, Laetitia Guillemette, Julie Ménard, Jean-Luc Ardilouze, Patrice Perron, Marie-France Hivert

**Affiliations:** Department of Medicine, Université de Sherbrooke, 3001 12th Avenue North, Sherbrooke, Québec Canada; Centre de recherche du Centre Hospitalier Universitaire de Sherbrooke, 3001 12th Avenue North, wing 9, door 6, Sherbrooke, Québec Canada; Diabetes Center, Massachusetts General Hospital, 50 Staniford Street, Boston, MA USA; Department of Population Medicine, Harvard Medical School, Harvard Pilgrim Health Care Institute, 401 Park Drive, suite 401, Boston, MA USA

**Keywords:** Leptin, Pregnancy, Weight gain, Body mass index

## Abstract

**Background:**

Excessive gestational weight gain (GWG) is associated with adverse pregnancy outcomes. In non-pregnant populations, low leptin levels stimulate positive energy balance. In pregnancy, both the placenta and adipose tissue contribute to circulating leptin levels. We tested whether maternal leptin levels are associated with subsequent GWG and whether this association varies depending on stage of pregnancy and on maternal body mass index (BMI).

**Methods:**

This prospective cohort study included 675 pregnant women followed from 1^st^ trimester until delivery. We collected anthropometric measurements, blood samples at 1^st^ and 2^nd^ trimester, and clinical data until delivery. Maternal leptin was measured by ELISA (Luminex technology). We classified women by BMI measured at 1^st^ trimester: BMI < 25 kg/m^2^ = normal weight; 25 ≤ BMI < 30 kg/m^2^ = overweight; and BMI ≥ 30 kg/m^2^ = obese.

**Results:**

Women gained a mean of 6.7 ± 3.0 kg between 1^st^ and 2^nd^ trimester (mid pregnancy GWG) and 5.6 ± 2.5 kg between 2^nd^ and the end of 3^rd^ trimester (late pregnancy GWG). Higher 1^st^ trimester leptin levels were associated with lower mid pregnancy GWG, but the association was no longer significant after adjusting for % body fat (%BF; β = 0.38 kg per log-leptin; SE = 0.52; *P* = 0.46). Higher 2^nd^ trimester leptin levels were associated with greater late pregnancy GWG and this association remained significant after adjustment for BMI (β = 2.35; SE = 0.41; *P* < 0.0001) or %BF (β = 2.01; SE = 0.42; *P* < 0.0001). In BMI stratified analyses, higher 2^nd^ trimester leptin levels were associated with greater late pregnancy GWG in normal weight women (β = 1.33; SE = 0.42; *P* = 0.002), and this association was stronger in overweight women (β = 2.85; SE = 0.94; *P* = 0.003 – *P* for interaction = 0.05).

**Conclusions:**

Our results suggest that leptin may regulate weight gain differentially at 1^st^ versus 2^nd^ trimester of pregnancy: at 2^nd^ trimester, higher leptin levels were associated with greater subsequent weight gain – the opposite of its physiologic regulation in non-pregnancy – and this association was stronger in overweight women. We suspect the existence of a feed-forward signal from leptin in second half of pregnancy, stimulating a positive energy balance and leading to greater weight gain.

**Electronic supplementary material:**

The online version of this article (doi:10.1186/s12884-016-0842-y) contains supplementary material, which is available to authorized users.

## Background

Excessive gestational weight gain (GWG) and pre-pregnancy obesity are associated with higher risk of adverse pregnancy outcomes, such as macrosomia, gestational diabetes mellitus (GDM), preeclampsia, and caesarean delivery [1]. Several factors influence GWG, including quality of diet, physical activity levels, pre-pregnancy body mass index (BMI), maternal age and parity [2, 3]. The physiologic regulation of GWG is highly variable from one woman to another [4]; a few pregnancy-related hormones such as progesterone have been suggested to participate in GWG regulation [5] but most endogenous regulators are still unknown.

Leptin is an adipokine secreted mainly from adipocytes that circulates in proportion to white adipose tissue mass in non-pregnant individuals [6], reflecting energy stores in adipose tissue. Leptin is known for its role in energy homeostasis: monogenic leptin deficiency is associated with hyperphagia and morbid obesity in both animals and humans [7–9]. However, leptin resistance also appears to exist in obese individuals since higher endogenic leptin levels or exogenic leptin administration does not reduce weight in humans [10, 11]. In a physiologic framework, lower leptin levels are associated with increased weight gain in normal-weight young adults [12] and with greater weight regain after weight loss in obese individuals [13].

In pregnancy, the placenta is a substantial source of circulating leptin and levels increase throughout pregnancy [14, 15]. This seems counter-intuitive to leptin’s classic physiological role, as pregnancy should be a state where the energy signalling pathways favour a positive balance by increasing food intake. While we know that adiposity accumulation leads to higher leptin levels [15–19], how leptin may conversely act as regulator of subsequent weight gain in pregnancy is less understood. Current literature on this topic is sparse and inconclusive because of inconsistencies in study design – leptin levels measured before, during or after weight gain assessment – and incomplete adjustment for potential confounders [19–21]. Thus, we present here our prospective study designed to assess the associations between baseline leptin levels and subsequent GWG in a large population-based cohort of pregnant women, taking into account maternal adiposity status and period of the pregnancy.

## Methods

We recruited pregnant women at 1^st^ trimester (5–16 weeks) of pregnancy and followed them until delivery in the prospective cohort study Glycemic regulation in Gestation and Growth (Gen3G). Pregnant women arriving for their 1^st^ trimester blood sample visit between January 2010 and July 2013 were invited to participate in our study if they expected to deliver at the Centre Hospitalier Universitaire de Sherbrooke (CHUS). We excluded women who had any of the following criteria: age <18 years old, multiple pregnancy, pregestational diabetes (type 1 or 2) or diabetes discovered at 1^st^ trimester (criteria based on 2008 Canadian Diabetes Association guidelines [22]), drugs and/or alcohol abuse, uncontrolled endocrine disease, or other major medical conditions (for attrition and further details, see [23]). The CHUS human-research ethics committee approved the project and all women gave written informed consent before they were included in the study, in accordance with the Declaration of Helsinki.

During their 1^st^ visit, we collected demographics and baseline characteristics of participants, including maternal age, ethnic background, gestational age (confirmed by echography), parity, and personal and familial medical history. In order to collect data on lifestyle, we used validated questionnaires concerning nutrition and physical activity adapted from the Canadian Community Health Survey [24]. Anthropometric measurements including weight (kg; by a calibrated electronic scale, bare feet, in light clothing), height (m), BMI (kg/m^2^), % body fat (%BF; estimated by electrical bioimpedance using a standing foot-to-foot scale) and systolic and diastolic blood pressures were performed according to standardized procedures as described previously [25]. The majority of women (97%) also performed a 50g glucose challenge test with glucose levels measured 1-h after glucose ingestion, for early screening of GDM. We collected random non-fasting blood samples in the remaining participants.

At 2^nd^ trimester (23–30 weeks), all women performed a 75g oral glucose tolerance test (OGTT) under fasting state for routine GDM diagnosis based on International Association of Diabetes and Pregnancy Study Groups criteria [26]. We collected extra fasting blood samples and at 1-h and 2-h during the OGTT for further analyses. Measures of glucose and insulin over the course of the OGTT allowed determination of dynamic indices of insulin sensitivity and secretion, respectively; Matsuda index (validated in pregnancy [27]), 10,000/[square root (fasting glucose × fasting insulin) × (mean glucose × mean insulin)] [28]; and total area under the curve (AUC)_insulin/glucose_ was calculated using the trapezoidal rule applied to the insulin and glucose curves during the OGTT. We collected data concerning any medical updates since the last visit and repeated the nutrition and physical activity questionnaires. Once again, we measured anthropometry, according to the same standardized procedures. We calculated maternal GWG (kg) between 1^st^ and 2^nd^ trimester (mid pregnancy GWG) by subtracting 1^st^ trimester from 2^nd^ trimester maternal measured weights.

At the end of the 3^rd^ trimester, we collected maternal weight (kg) based on the last prenatal visit (34–42 weeks of gestation) available in our electronic medical records (EMR). From the EMR, we additionally collected medical events from 2^nd^ trimester to delivery for any additional major complications (premature deliveries, GDM, and pre-eclampsia) and placental and birth weights recorded by clinical staff at delivery. Women lost to follow-up did not differ from the ones followed until delivery [23]. We calculated maternal weight gain between 2^nd^ and 3^rd^ trimester (late pregnancy GWG) by subtracting 2^nd^ trimester from 3^rd^ trimester maternal weights.

### Laboratory measurements

All blood samples collected at 1^st^ and 2^nd^ trimester were maintained at 4°C and centrifuged. Plasma was distributed in aliquots and stored at −80°C until measurements. Plasma leptin and insulin levels were measured by enzyme-linked immunosorbent assay (ELISA Luminex technology; Millipore Corp, Billerica, MA, USA). Plasma glucose levels were measured by glucose hexokinase (Roche Diagnostics, Indianapolis, IN, USA). Intra- and inter-assays coefficients of variation for leptin levels were respectively of 3 and 4%.

### Statistical analyses

From all women followed until delivery, we excluded pregnancies with major complications (prematurity <37 weeks, pre-eclampsia) and missing data for weight or leptin levels at any of the time points. Participants excluded for complications, missing data or lost to follow-up (*n* = 288) were similar to women remaining in our dataset (*n* = 736) in terms of maternal age, ethnicity and pre-pregnancy BMI (all *P* > 0.05). We further excluded women diagnosed with GDM (*n* = 61) for this analysis because diagnosis and treatment of GDM greatly influence GWG. This report included 675 normoglycemic pregnant women who had a complete dataset from 1^st^ trimester to delivery, including all necessary maternal weight and leptin levels.

Characteristics of participants are presented as means ± standard deviations if normally distributed or as median and interquartile ranges otherwise; categorical variables are presented as percentage. Continuous variables not normally distributed were log-transformed and we used log-transformed variables for correlations and linear regression models when appropriate; leptin levels were normally distributed after log-transformation. We performed correlation analyses (Pearson and Spearman as appropriate) to assess variables associated cross-sectionally with leptin levels and to evaluate potential confounders. We conducted linear regression analyses to assess associations between leptin levels (per one log increase) and subsequent GWG (using kg/week to standardize for different follow-up time), taking into account potential confounding factors. We first adjusted for gestational weeks and BMI (model 2a) measured cross-sectionally with leptin levels of interest at each time period assessed: for example the model testing the association between leptin at 1^st^ trimester and subsequent GWG was adjusted for gestational age and BMI measured at 1^st^ trimester. We then adjusted for %BF instead of BMI to account for adiposity per se (model 3a). We additionally adjusted for systolic and diastolic blood pressures, physical activity, quantity of fruits and vegetables consumed per day and frequency of restaurant meals per week to account for potential confounders including lifestyle (models 2b and 3b) using characteristics measured cross-sectionally with leptin levels for each respective model. We also performed linear regression analyses according to initial 1^st^ trimester BMI categories as internationally defined (BMI <25 kg/m^2^ = normal weight; 25 ≤ BMI <30 kg/m^2^ = overweight; or BMI ≥30 kg/m^2^ = obese) to assess associations between leptin levels (per one log increase) and subsequent GWG (kg/week) per clinically defined BMI status. In addition to linear regression analyses assessing GWG per week (in tables), we performed subsidiary linear regression analyses with weight gain expressed as GWG between 1^st^ and 2^nd^ trimester (mid pregnancy GWG) and GWG between 2^nd^ and 3^rd^ trimester (late pregnancy GWG) for easier interpretation. We also conducted sensitivity analyses: 1- excluding underweight women defined as 1^st^ trimester BMI <18.5 kg/m^2^ (*n* = 17); 2- excluding morbidly obese women defined as 1^st^ trimester BMI >40 kg/m^2^ (*n* = 9); and by adding an additional variable in adjusted models, namely: 3- placental weight (available in 529 deliveries); 4- Matsuda index (insulin sensitivity index); or 5- AUC_insulin/glucose_ (insulin secretion index). *P* < 0.05 was considered statistically significant. All the analyses were performed using version 18 of Statistical Package for the Social Sciences (SPSS) for Windows.

## Results

Characteristics of the 675 pregnant women included in the present study are presented in Table 1. Participants were 28.2 ± 4.3 years old and 97.2% were of European descent, similar to the general population of pregnant women receiving care at our institution [23].

**Table 1:** Characteristics of 675 pregnant women included in Gen3G at 1^st^ and 2^nd^ trimesters

Characteristics	1^st^ trimester	2^nd^ trimester
	Mean ± SD or median [IQR] or %	Mean ± SD or median [IQR] or %
Age (years)	28.2 ± 4.3	---
Ethnic background	97.2	---
(% European descent)		
Parity (% primiparous)	52.0	---
Gestational weeks	9.4 ± 2.0	26.4 ± 1.0
Body mass index (kg/m^2^)	23.9 [21.5–27.3]	26.6 [24.1–29.8]
% body fat	31.3 ± 8.0	35.4 ± 6.4
Systolic blood pressure (mmHg)	110.5 ± 9.8	107.3 ± 8.9
Diastolic blood pressure (mmHg)	68.9 ± 6.9	67.4 ± 6.6
Physical activity (kcal/kg/day)	1.1 [0.5–1.9]	0.9 [0.4–1.5]
Nutrition		
Fruits & vegetables (per day)	5.6 ± 2.4	6.1 ± 2.5
Restaurant meals (per week)	1.0 [0.5–2.0]	1.0 [0.5–2.0]
Leptin levels non fasting (ng/ml)	8.0 [4.5–13.0]	---
Leptin levels during OGTT (ng/ml)		
Fasting	---	13.3 [8.3–20.6]
1-h post OGTT	---	10.3 [6.1–16.0]
2-h post OGTT	---	9.6 [5.3–15.3]

Data are presented as mean ± standard deviation (SD) or median (interquartile range). Variables not normally distributed were log-transformed. At 1^st^ trimester, ranges of leptin levels were 0.9 to 59.7 ng/ml. At 2^nd^ trimester, ranges of leptin were 1.1 to 85.8 ng/ml fasting, 0.9 to 56.2 ng/ml 1-h post OGTT, and 0.8 to 53.2 ng/ml 2-h post OGTT. For ethnic background and % body fat at 1^st^ trimester: *n* = 671. For gestational weeks at 2^nd^ trimester and body mass index at 2^nd^ trimester: *n* = 674. For parity and systolic and diastolic blood pressures at 2^nd^ trimester: *n* = 673. For waist circumference at 1^st^ trimester: *n* = 662. For physical activity at 1^st^ trimester: *n* = 666. For nutrition: 641 ≤ n ≤ 667. For % body fat at 2^nd^ trimester and physical activity at 2^nd^ trimester: *n* = 669

### Adiposity and weight gain

At 1^st^ trimester, participants had a median BMI of 23.9[21.5–27.3] kg/m^2^ and a mean %BF equal to 31.3 ± 8.0 % (see Table 1). At 2^nd^ trimester, median BMI was 26.6[24.1–29.8] kg/m^2^ and mean %BF was 35.4 ± 6.4 %. Women gained a mean of 6.7 ± 3.0 kg between 1^st^ and 2^nd^ trimester (weekly GWG = 0.40 ± 0.18 kg/week), and a mean of 5.6 ± 2.5 kg between 2^nd^ trimester and late 3rd trimester (weekly GWG = 0.54 ± 0.23 kg/week) for a global mean GWG of 12.2 ± 4.4 kg between the 1^st^ and the end of the 3^rd^ trimester. Maternal factors at 1^st^ trimester associated with larger subsequent GWG were lower adiposity levels (lower BMI and %BF) and being assessed at an earlier point of the pregnancy (as represented by gestational weeks). Other factors measured at 1^st^ or 2^nd^ trimester were weakly or non-significantly associated with subsequent GWG (see Additional file 1: Table S1). At delivery, birth weight was 3.408 ± 0.461 kg and placental weight was 557.4 ± 133.4 g in average. Most maternal weight and adiposity variables at 1^st^ and 2^nd^ trimester were positively correlated with birth weight and placental weight; the strongest associations were observed between maternal weight or %BF and either birth outcomes (see Additional file 2: Table S2).

### Leptin levels

Leptin levels increased from 1^st^ (8.0[4.5–13.0] ng/ml) to 2^nd^ trimester (10.3[6.1–16.0] ng/ml 1-h post OGTT; see Table 1), with a increase between 1^st^ and 2^nd^ trimester of 2.1[−0.5–5.3] ng/ml. Maternal factors positively associated cross–sectionally with leptin levels at 1^st^ and 2^nd^ trimester were adiposity levels (BMI and %BF) and blood pressures, while other factors were weakly or non-significantly associated with leptin levels (see Table 2 and Additional file 3: Table S3). Birth weight and placental weight were also weakly associated with maternal leptin levels at 1^st^ and 2^nd^ trimester (see Additional file 2: Table S2); these associations were substantially reduced and none of them remained significant after adjustments for maternal BMI.

**Table 2:** Cross-sectional correlations between women’s characteristics and leptin levels measured at 1^st^ and 2^nd^ trimester

Characteristics at 1^st^ trimester	Correlations^a^ with leptin levels at 1^st^ trimester	
	r	*P* value
Age (years)	0.04	0.31
Gestational weeks	0.12	0.002
Body mass index (kg/m^2^)	0.73	<0.0001
% body fat	0.74	<0.0001
Systolic blood pressure (mmHg)	0.21	<0.0001
Diastolic blood pressure (mmHg)	0.25	<0.0001
Physical activity (kcal/kg/day)	−0.11	0.005
Nutrition		
Fruits & vegetables (per day)	−0.09	0.02
Restaurant meals (per week)	0.10	0.01
Characteristics at 2^nd^ trimester	Correlations^a^ with leptin levels 1-h post OGTT at 2^nd^ trimester	
	r	*P* value
Gestational weeks	−0.04	0.25
Body mass index (kg/m^2^)	0.67	<0.0001
% body fat	0.69	<0.0001
Systolic blood pressure (mmHg)	0.23	<0.0001
Diastolic blood pressure (mmHg)	0.31	<0.0001
Physical activity (kcal/kg/day)	−0.07	0.08
Nutrition		
Fruits & vegetables (per day)	−0.09	0.02
Restaurant meals (per week)	0.09	0.02

^a^These are all Pearson correlations, except for correlations with physical activity at 1^st^ and 2^nd^ trimester that are Spearman correlations

### Leptin levels and subsequent weight gain

Table 3 presents linear regression analyses testing the associations between leptin levels and subsequent GWG. In unadjusted analyses, higher 1^st^ trimester leptin levels were associated with lower mid pregnancy GWG (β = −1.68, meaning a decrease of 1.68 kg per increase of one log-leptin at 1^st^ trimester; standard error (SE) = 0.35; *P* < 0.0001). After adjusting for 1^st^ trimester gestational weeks and BMI (model 2a), the direction of effect was reversed, meaning that higher leptin levels were associated with greater GWG (β = 1.32; SE = 0.49; *P* = 0.007). Replacing BMI by %BF at 1^st^ trimester (model 3a) showed flattening of the association and leptin levels were no longer associated with mid pregnancy GWG (β = 0.38; SE = 0.52; *P* = 0.46). Further adjustments for blood pressure, physical activity, fruit and vegetable portions per day and restaurant meals per week did not modify associations in BMI of %BF adjusted models.

**Table 3:** Associations between 1^st^ or 2^nd^ trimester leptin levels and subsequent mid or late pregnancy GWG^a^

Models	Associations: 1^st^ trimester leptin levels and mid pregnancy GWG		Associations: 2^nd^ trimester leptin levels 1-h post OGTT and late pregnancy GWG	
	β ± SE	*P* value	β ± SE	*P* value
Model 1: unadjusted	−0.08 ± 0.02	<0.0001	0.11 ± 0.03	0.0001
Model 2a: adjusted for BMI and gestational weeks	0.07 ± 0.03	0.01	0.24 ± 0.04	<0.0001
Model 2b: BMI-fully adjusted^1^	0.07 ± 0.03	0.02	0.21 ± 0.04	<0.0001
Model 3a: adjusted for %BF and gestational weeks	0.01 ± 0.03	0.64	0.20 ± 0.04	<0.0001
Model 3b: %BF-fully adjusted^1^	0.01 ± 0.03	0.74	0.18 ± 0.04	<0.0001

^a^All β represent the change in weight gain (kg) per week associated to a change of 1 log of leptin levels. GWG: gestational weight gain. BMI: body mass index. %BF: percent body fat. OGTT: oral glucose tolerance test. All models were adjusted with variables measured cross-sectionally with leptin levels of interest. For example in model 2a, model testing association between leptin levels measured at 1^st^ trimester and mid pregnancy GWG is adjusted for BMI at 1^st^ trimester and the number of weeks of gestation at the moment of assessment. ^1^Adjusted for further potential confounders (always measured cross-sectionally to leptin levels respectively to trimester): systolic and diastolic blood pressures (1^st^ or 2^nd^ trimester), physical activity (1^st^ or 2^nd^ trimester), fruits and vegetables per day (1^st^ or 2^nd^ trimester) and restaurant meals per week (1^st^ or 2^nd^ trimester)

Higher 2^nd^ trimester leptin levels were associated with greater late pregnancy GWG (β = 1.17; SE = 0.31; *P* = 0.0002 for 1-h post OGTT leptin levels). Associations were strengthened by adjustment for 2^nd^ trimester gestational weeks and BMI with effect sizes almost doubling (β = 2.35; SE = 0.41; *P* < 0.0001 again for 1-h post OGTT leptin levels). Replacing BMI by %BF did not modify results in size or direction of effect. All associations remained statistically significant after further adjustment for blood pressure, physical activity, fruit and vegetable portions per day and restaurant meals per week, all measured at 2^nd^ trimester (β = 2.15; SE = 0.42; *P* < 0.0001 for 1-h post OGTT leptin levels). Excluding underweight women (BMI <18.5 kg/m^2^) or morbidly obese women (BMI >40 kg/m^2^), or adding placental weight as an additional co-variable did not modify the results in sensitivity analyses. Our sensitivity analyses including insulin sensitivity or insulin secretion indices did not modify the associations. Linear regression analyses concerning late pregnancy GWG and 2^nd^ trimester leptin levels measured fasting and 2-h post OGTT showed similar associations than those observed with 2^nd^ trimester 1-h post OGTT leptin levels (see Additional file 4: Table S4).

### Leptin levels and subsequent weight gain stratified by maternal weight status

In order to further investigate the role of initial adiposity in the association between leptin levels and subsequent GWG, we performed our analyses stratified by maternal weight status defined by BMI measured at 1^st^ trimester (see Table 4). We found no association between 1^st^ trimester leptin levels and mid pregnancy GWG in any BMI strata, neither in unadjusted nor in multivariate models (all *P* > 0.05).

**Table 4:** Associations between 1^st^ or 2^nd^ trimester leptin and subsequent GWG^a^ stratified by 1^st^ trimester BMI

Models		BMI < 25 kg/m^2^ (*n* = 406)		25 ≤ BMI < 30 kg/m^2^ (*n* = 159)		BMI ≥ 30 kg/m^2^ (*n* = 110)	
		β ± SE	*P* value	β ± SE	*P* value	β ± SE	*P* value
1^st^ trimester leptin levels and mid pregnancy GWG	Unadjusted	0.02 ± 0.03	0.46	0.10 ± 0.08	0.23	0.01 ± 0.09	0.92
	Adjusted^1^	0.01 ± 0.03	0.60	0.01 ± 0.08	0.88	−0.01 ± 0.10	0.92
2^nd^ trimester leptin levels 1-h post OGTT and late pregnancy GWG	Unadjusted	0.17 ± 0.04	<0.0001	0.33 ± 0.08	<0.0001	0.12 ± 0.12	0.33
	Adjusted^1^	0.15 ± 0.04	0.0001	0.26 ± 0.09	0.004	0.13 ± 0.12	0.29

^a^All β represent the change in weight gain (kg) per week associated to a change of 1 log of leptin levels. ^1^ Adjusted for variables measured cross-sectionally to leptin levels of interest respectively to trimester: the number of weeks of gestation at the moment of assessment, systolic and diastolic blood pressures (1^st^ or 2^nd^ trimester), physical activity (1^st^ or 2^nd^ trimester), fruits and vegetables per day (1^st^ or 2^nd^ trimester) and restaurant meals per week (1^st^ or 2^nd^ trimester). BMI: body mass index. GWG: gestational weight gain. OGTT: oral glucose tolerance test

Higher 2^nd^ trimester leptin levels were associated with greater late pregnancy GWG in normal weight women (unadjusted β = 1.66, kg per log-leptin levels at 1h-OGTT; SE = 0.41; *P* < 0.0001). Associations between leptin levels and late GWG appeared stronger among overweight women with effect sizes almost twice the ones observed in normal weight women (unadjusted β = 3.60; SE = 0.88; *P* < 0.0001 – *P* = 0.05 for interaction between normal weight and overweight strata). Strength of associations was slightly reduced by adding potential confounders in fully adjusted models for normal weight and overweight women, mainly driven by presence of blood pressure and consumption of fruits/vegetables in the models. We observed no significant associations between 2^nd^ trimester leptin levels and late pregnancy GWG in obese women (unadjusted β = 0.85; SE = 1.29; *P* = 0.51 for 1-h post OGTT leptin levels) in either unadjusted or adjusted analyses. After removing women with BMI >40 kg/m^2^ from the obese group, we observed that the effect size was slightly greater (unadjusted β = 1.66; SE = 1.38; *P* = 0.23 for 1-h post OGTT leptin levels; *n* = 101 women) but this remained non-significant. Our sensitivity analyses excluding underweight women (BMI <18.5 kg/m^2^) or including either placental weight, insulin sensitivity index or insulin secretion index as co-variables did not modify the results. Linear regression analyses using 2^nd^ trimester leptin levels measured fasting and 2-h post OGTT showed similar associations than those observed with 1-h post OGTT leptin levels (see Additional file 5: Table S5).

## Discussion

We demonstrated that higher 2^nd^ trimester leptin levels were associated with greater subsequent GWG in pregnant women from a general population cohort, independent of maternal adiposity and other confounders. This positive association is the opposite of the expected physiologic regulation observed in non-pregnant individuals. Interestingly, this positive association was particularly observable and stronger in women classified as overweight at the beginning of pregnancy than in normal weight women.

Only a few studies have reported associations between baseline maternal leptin levels – measured at the beginning of the weight gain period – and subsequent GWG [19–21]. Our findings at 1^st^ trimester are in line with previous findings from Walsh et al. and Kim et al., where 1^st^ trimester leptin levels do not show a significant association with subsequent GWG after adjustments for %BF and other confounders [19, 21]. Thus, based on our results and those of others, lower 1^st^ trimester leptin levels represent lower adiposity, signaling the need to increase positive energy balance and leading to greater weight gain early in pregnancy – similar to non-pregnant leptin’s role into central nervous system (CNS) signaling.

Our study adds to the current literature by investigating leptin levels measured at 24–28 weeks and subsequent GWG in a wide range of maternal BMI status. In contrast to our results, Kim et al. did not show associations between leptin levels measured at 24 weeks and any weight variables, including GWG. Difference in populations or limited power due to a smaller sample size (*n* = 75) may explain different findings from Kim et al. [21]. Walsh et al. measured leptin at 28 weeks, but assessed association with overall GWG throughout pregnancy – i.e. spanning a time period before and after our leptin measurement, and thus, we cannot compare their results to ours [19]. In line with our results in normal weight pregnant women, Stein et al. reported that higher leptin levels measured at 17 weeks of gestation were associated with a higher measured rate of weight gain between 20 and 36 gestational weeks in women with a pregravid BMI from 19.8 to 26.0 kg/m^2^ [20]. Our results expand current knowledge and suggest that this positive association is more prominent in overweight women. This is highly intriguing, as we know that clinically, overweight women are more likely to gain excessive GWG compared to normal weight and obese women.

The physiologic role of leptin during pregnancy is still obscure, but our results suggest that leptin promotes weight gain in a feed-forward mechanism during the second half of pregnancy. The placenta produces leptin in high quantity and contributes to maternal circulating levels throughout pregnancy, but why the placenta produces such a high quantity of leptin remains in question. Our results showing larger effect size in overweight women argue for the existence of this feed-forward regulation, as overweight women presented higher levels of leptin throughout pregnancy: if a pregnancy-specific CNS signal emerges as pregnancy advances, rising placental-derived leptin, in addition to high leptin from pre-pregnancy overweight status, might enhanced this positive feed-forward loop. Our findings of reduced associations in obese women could be explained by a state of central leptin resistance when levels reach the extreme upper range; but they could also reflect obese women self-regulating their energy balance based on clinical recommendations. It is also possible that leptin resistance occurring at the placenta level contributes to the absence of this feed-forward mechanism in obese women [29, 30]. However, the reduced effect sizes we observed in obese women should be interpreted with caution, given large confidence intervals and our smaller sample in this stratum.

Animal studies have supported the existence of a pregnancy-induced leptin resistance status [31–34]. For example, Ladyman et al. observed that intraperitoneal injections of leptin did not suppress food intake in pregnant mice compared to its observed effect of food intake reduction in non-pregnant mice [31]. The same report also demonstrated that the ventromedial hypothalamus and the paraventricular nucleus of pregnant mice showed a decrease in leptin sensitivity compared to non-pregnant mice [31]. Based on animal models, other potential mechanisms that could contribute to leptin resistance during pregnancy include an increase in circulating leptin binding protein, an impairment in leptin transport through the blood brain barrier, a decrease in Ob-Rb (isoform b of leptin’s receptor) mRNA (messenger ribonucleic acid) levels in the hypothalamus, and/or an impairment in the intracellular signalization induced by the binding of leptin to Ob-Rb [32–34]. In term human placenta tissue sections from obese pregnancies, it has also been shown that Ob-Rb is down regulated in comparison to lean pregnancies, raising the possibility of placental leptin resistance [29, 30]. Our results add to the current knowledge in suggesting that a state of leptin resistance also exists in human pregnancy, though the exact mechanisms leading to pregnancy-induced leptin resistance and a possible feed-forward loop in humans remain to be investigated. It did not seem that insulin resistance or insulin secretion influenced our observations, but other factors such as specific pregnancy hormones may be implicated and merit future investigations.

### Strengths and limitations

A major strength of our study is the design, as participants were followed prospectively and represent the general population of pregnant women receiving care at our hospital [35]. Strengths of this study also include our large sample size, exclusion of diabetes at 1^st^ and 2^nd^ trimester, and use of standardized procedures with high reliability for all laboratory and clinical measures, including anthropometry.

The main limitation of our study is the observational study design; consequently, we cannot conclude about causality or any mechanistic pathways. Despite being validated in pregnancy [36], measurement of %BF by bioimpedance could be influenced differently at 2^nd^ trimester by the presence of larger amount of body water, the placenta and the fetus. We used validated questionnaires for self-report of physical activity and nutrition, but direct measurements of energy intake and expenditure would have provided greater precision. Our population is mainly of European descent, so our results might not apply to other ethnicities.

## Conclusions

In summary, our results suggest that body weight regulation by leptin during the second half of pregnancy does not follow the physiologic role as an adipostat with negative feedback to CNS that is classically attributed to leptin. We revealed a positive association between higher 2^nd^ trimester leptin levels and greater subsequent GWG in normal weight women, and this association was stronger in overweight women. Our findings suggest a potential feed forward mechanism where higher leptin levels could signal the need to increase food intake and lead to greater weight gain. Feed forward loops are rare in human physiology, but are well known in the reproductive endocrine system – such as the menstrual cycle [37]. Mechanistic studies will be necessary to test this hypothesis and elucidate these mechanisms at the CNS and peripheral levels.

## Ethics approval and consent to participate

The Centre Hospitalier Universitaire de Sherbrooke human-research ethics committee approved the project and all women gave a written informed consent before they were included in the study, in accordance with the Declaration of Helsinki.

## Consent for publication

Not applicable.

## Availability of data and materials

The dataset supporting the conclusions of this article is available upon request.

## Additional files

Additional file 1: Table S1. Correlations between women’s characteristics and subsequent GWG (expressed per week) in mid and late pregnancy. (DOCX 27 kb) Additional file 2: Table S2. Birth and placenta weights correlations with maternal weight-related variables and maternal leptin levels during pregnancy. (DOCX 27 kb) Additional file 3: Table S3. Correlations between women’s characteristics and leptin levels measured at 2^nd^ trimester. (DOCX 27 kb) Additional file 4: Table S4. Correlations between 2^nd^ trimester leptin levels and subsequent GWG (expressed per week)*. (DOCX 27 kb) Additional file 5: Table S5. Correlations between 2^nd^ trimester leptin levels and subsequent GWG* stratified by 1^st^ trimester BMI. (DOCX 27 kb)

## Abbreviations

%BF: percent body fat
AUC: area under the curve
BMI: body mass index
CHUS: Centre Hospitalier Universitaire de Sherbrooke
CNS: central nervous system
ELISA: enzyme-linked immunosorbent assay
EMR: electronic medical records
GDM: gestational diabetes mellitus
GWG: gestational weight gain
mRNA: messenger ribonucleic acid
Ob-Rb: isoform b of leptin’s receptor
OGTT: oral glucose tolerance test
SD: standard deviation
SE: standard error
SPSS: Statistical Package for the Social Sciences
