# Supplementary material for: Higher maternal leptin levels at second trimester are associated with subsequent greater gestational weight gain in late pregnancy
Source: BMC Pregnancy Childbirth. 2016 Mar 22;16:62. doi: 10.1186/s12884-016-0842-y (PMC4802837; doi:10.1186/s12884-016-0842-y)
Supplement: Additional file 1: Table S1. — Correlations between women’s characteristics and subsequent GWG (expressed per week) in mid and late pregnancy. (DOCX 27 kb) [file 12884_2016_842_MOESM1_ESM.docx]

Table S1 – Correlations between women’s characteristics and subsequent GWG (expressed per week) in mid and late pregnancy

| Characteristics | Correlations* with weight gain between **1^st^ and 2^nd^ trimesters** | | Correlations with weight gain between **2^nd^ trimester and delivery** | |
| --- | --- | --- | --- | --- |
|  | r | *P* value | r | *P* value |
| **1^st^ trimester** |  |  |  |  |
| Age (years) | 0.02 | 0.62 |  |  |
| Gestational weeks | 0.14 | 0.0004 |  |  |
| Body mass index (kg/m^2^) | -0.31 | <0.0001 |  |  |
| % body fat | -0.24 | <0.0001 |  |  |
| Systolic blood pressure (mmHg) | -0.10 | 0.007 |  |  |
| Diastolic blood pressure (mmHg) | -0.11 | 0.006 |  |  |
| Physical activity (kcal/kg/day) | -0.01 | 0.88 |  |  |
| Nutrition |  |  |  |  |
| Fruits & vegetables (per day) | 0.04 | 0.28 |  |  |
| Restaurant meals (per week) | 0.08 | 0.04 |  |  |
| **2^nd^ trimester** |  |  |  |  |
| Gestational weeks |  |  | 0.05 | 0.20 |
| Body mass index (kg/m^2^) |  |  | -0.04 | 0.30 |
| % body fat |  |  | 0.01 | 0.77 |
| Systolic blood pressure (mmHg) |  |  | 0.04 | 0.36 |
| Diastolic blood pressure (mmHg) |  |  | 0.06 | 0.12 |
| Physical activity (kcal/kg/day) |  |  | -0.08 | 0.03 |
| Nutrition |  |  |  |  |
| Fruits & vegetables (per day) |  |  | -0.02 | 0.68 |
| Restaurant meals (per week) |  |  | 0.05 | 0.18 |

* These are all Pearson correlations, except for correlations with physical activity at 1^st^ and 2^nd^ trimesters that are Spearman correlations.
