# Supplementary material for: Higher maternal leptin levels at second trimester are associated with subsequent greater gestational weight gain in late pregnancy
Source: BMC Pregnancy Childbirth. 2016 Mar 22;16:62. doi: 10.1186/s12884-016-0842-y (PMC4802837; doi:10.1186/s12884-016-0842-y)
Supplement: Additional file 2: Table S2. — Birth and placenta weights correlations with maternal weight-related variables and maternal leptin levels during pregnancy. (DOCX 27 kb) [file 12884_2016_842_MOESM2_ESM.docx]

Table S2 –Birth and placenta weights correlations with maternal weight-related variables and maternal leptin levels during pregnancy

|  | Correlations* with | | | |
| --- | --- | --- | --- | --- |
|  | Birth weight | | Placental weight | |
|  | r | *P* value | r | *P* value |
| Characteristics at **1^st^ trimester** |  |  |  |  |
| Weight (kg) | 0.26 | <0.0001 | 0.23 | <0.0001 |
| Body mass index (kg/m^2^) | 0.17 | <0.0001 | 0.19 | <0.0001 |
| % body fat | 0.23 | <0.0001 | 0.20 | <0.0001 |
| Leptin levels (ng/ml) | 0.14 | 0.0002 | 0.13 | 0.004 |
| Weight gain per week between 1^st^ and 2^nd^ trimesters (kg) | 0.13 | 0.0007 | 0.03 | 0.42 |
| Characteristics at **2^nd^ trimester** |  |  |  |  |
| Weight (kg) | 0.30 | <0.0001 | 0.26 | <0.0001 |
| Body mass index (kg/m^2^) | 0.20 | <0.0001 | 0.22 | <0.0001 |
| % body fat | 0.25 | <0.0001 | 0.22 | <0.0001 |
| Leptin levels fasting (ng/ml) | 0.13 | 0.0006 | 0.17 | <0.0001 |
| Leptin levels 1-h post OGTT (ng/ml) | 0.11 | 0.003 | 0.12 | 0.008 |
| Leptin levels 2-h post OGTT (ng/ml) | 0.11 | 0.004 | 0.12 | 0.005 |
| Weight gain per week between 2^nd^ trimester and delivery (kg) | 0.08 | 0.05 | 0.12 | 0.006 |
| Characteristics at the **end of 3rd trimester** |  |  |  |  |
| Weight (kg) | 0.31 | <0.0001 | 0.28 | <0.0001 |
| Weight gain per week between 1^st^ trimester and delivery (kg) | 0.14 | 0.0004 | 0.09 | 0.03 |

* These are all Pearson correlations.
