# Supplementary material for: Higher maternal leptin levels at second trimester are associated with subsequent greater gestational weight gain in late pregnancy
Source: BMC Pregnancy Childbirth. 2016 Mar 22;16:62. doi: 10.1186/s12884-016-0842-y (PMC4802837; doi:10.1186/s12884-016-0842-y)
Supplement: Additional file 3: Table S3. — Correlations between women’s characteristics and leptin levels measured at 2nd trimester. (DOCX 27 kb) [file 12884_2016_842_MOESM3_ESM.docx]

Table S3 – Correlations between women’s characteristics and leptin levels measured at 2^nd^ trimester

| Characteristics at 2^nd^ trimester | Correlations with leptin levels at 2^nd^ trimester | | | | | |
| --- | --- | --- | --- | --- | --- | --- |
|  | Fasting | | 1-h post OGTT | | 2-h post OGTT | |
|  | r | *P* value | r | *P* value | r | *P* value |
| Gestational weeks | -0.05 | 0.22 | -0.04 | 0.25 | -0.05 | 0.18 |
| Body mass index (kg/m^2^) | 0.65 | <0.0001 | 0.67 | <0.0001 | 0.68 | <0.0001 |
| % body fat | 0.65 | <0.0001 | 0.69 | <0.0001 | 0.69 | <0.0001 |
| Systolic blood pressure (mmHg) | 0.23 | <0.0001 | 0.23 | <0.0001 | 0.23 | <0.0001 |
| Diastolic blood pressure (mmHg) | 0.31 | <0.0001 | 0.31 | <0.0001 | 0.31 | <0.0001 |
| Physical activity (kcal/kg/day) | -0.07 | 0.08 | -0.07 | 0.08 | -0.07 | 0.06 |
| Nutrition |  |  |  |  |  |  |
| Fruits & vegetables (per day) | -0.12 | 0.002 | -0.09 | 0.02 | -0.12 | 0.002 |
| Restaurant meals (per week) | 0.09 | 0.02 | 0.09 | 0.02 | 0.11 | 0.007 |

* These are all Pearson correlations, except for correlations with physical activity that are Spearman correlations.
