# Supplementary material for: Higher maternal leptin levels at second trimester are associated with subsequent greater gestational weight gain in late pregnancy
Source: BMC Pregnancy Childbirth. 2016 Mar 22;16:62. doi: 10.1186/s12884-016-0842-y (PMC4802837; doi:10.1186/s12884-016-0842-y)
Supplement: Additional file 4: Table S4. — Correlations between 2nd trimester leptin levels and subsequent GWG (expressed per week)*. (DOCX 27 kb) [file 12884_2016_842_MOESM4_ESM.docx]

Table S4 – Correlations between 2^nd^ trimester leptin levels and subsequent GWG (expressed per week)*

| Models | Correlations: late pregnancy GWG and | | | | | |
| --- | --- | --- | --- | --- | --- | --- |
|  | 2^nd^ trimester leptin levels fasting | | 2^nd^ trimester leptin levels 1-h post OGTT | | 2^nd^ trimester leptin levels 2-h post OGTT | |
|  | β ± SE | *P* value | β ± SE | *P* value | β ± SE | *P* value |
| Model 1: unadjusted | 0.08 ± 0.03 | 0.006 | 0.11 ± 0.03 | 0.0001 | 0.09 ± 0.03 | 0.0008 |
| Model 2a: adjusted for BMI and gestational weeks | 0.18 ± 0.04 | <0.0001 | 0.24 ± 0.04 | <0.0001 | 0.21 ± 0.04 | <0.0001 |
| Model 2b: BMI-fully adjusted^1^ | 0.16 ± 0.04 | 0.0001 | 0.21 ± 0.04 | <0.0001 | 0.18 ± 0.04 | <0.0001 |
| Model 3a: adjusted for %BF and gestational weeks | 0.14 ± 0.04 | 0.0007 | 0.20 ± 0.04 | <0.0001 | 0.17 ± 0.04 | <0.0001 |
| Model 3b: %BF-fully adjusted^1^ | 0.12 ± 0.04 | 0.005 | 0.18 ± 0.04 | <0.0001 | 0.15 ± 0.04 | 0.0001 |

* All β represent the change in weight gain (kg) per week associated to a change of 1 log of leptin levels. GWG: gestational weight gain. BMI: body mass index. %BF: percent body fat. OGTT: oral glucose tolerance test. ^1^ Adjusted for further potential confounders: systolic and diastolic blood pressures, physical activity, fruits and vegetables per day and restaurant meals per week. All models were adjusted with variables measured at 2^nd^ trimester.
