# Supplementary material for: Higher maternal leptin levels at second trimester are associated with subsequent greater gestational weight gain in late pregnancy
Source: BMC Pregnancy Childbirth. 2016 Mar 22;16:62. doi: 10.1186/s12884-016-0842-y (PMC4802837; doi:10.1186/s12884-016-0842-y)
Supplement: Additional file 5: Table S5. — Correlations between 2nd trimester leptin levels and subsequent GWG* stratified by 1st trimester BMI. (DOCX 27 kb) [file 12884_2016_842_MOESM5_ESM.docx]

Table S5 – Correlations between 2^nd^ trimester leptin levels and subsequent GWG* stratified by 1^st^ trimester BMI

| Late pregnancy GWG and 2^nd^ trimester leptin levels | | BMI < 25 kg/m^2^  (n = 406) | | 25 ≤ BMI < 30 kg/m^2^  (n = 159) | | BMI ≥ 30 kg/m^2^  (n = 110) | |
| --- | --- | --- | --- | --- | --- | --- | --- |
|  |  | β ± SE | *P* value | β ± SE | *P* value | β ± SE | *P* value |
| Fasting | Unadjusted | 0.14 ± 0.04 | 0.0004 | 0.24 ± 0.09 | 0.009 | 0.13 ± 0.12 | 0.27 |
|  | Adjusted^1^ | 0.12 ± 0.04 | 0.003 | 0.13 ± 0.10 | 0.21 | 0.12 ± 0.12 | 0.33 |
| 1-h post OGTT | Unadjusted | 0.17 ± 0.04 | <0.0001 | 0.33 ± 0.08 | <0.0001 | 0.12 ± 0.12 | 0.33 |
|  | Adjusted^1^ | 0.15 ± 0.04 | 0.0001 | 0.26 ± 0.09 | 0.004 | 0.13 ± 0.12 | 0.29 |
| 2-h post OGTT | Unadjusted | 0.14 ± 0.04 | 0.0001 | 0.27 ± 0.08 | 0.0006 | 0.17 ± 0.11 | 0.13 |
|  | Adjusted^1^ | 0.12 ± 0.04 | 0.002 | 0.19 ± 0.08 | 0.02 | 0.17 ± 0.12 | 0.14 |

* All β represent the change in weight gain (kg) per week associated to a change of 1 log of leptin levels. GWG: gestational weight gain. BMI: body mass index. OGTT: oral glucose tolerance test. ^1^ Adjusted for 2^nd^ trimester variables: the number of weeks of gestation at the moment of assessment, systolic and diastolic blood pressures, physical activity, fruits and vegetables per day and restaurant meals per week.
